# Book Notices and Reviews

**Published:** 1864-04

**Authors:** 


### BOOK NOTICES AND REVIEWS.

*Proceedings of the American Pharmaceutical Association at its 11th Annual Meeting, Held in Baltimore, Md., Sept.,* 1863. *Also the Constitution and Roll of Members.*

This is a very interesting work of more than three hundred pages, issued by J. B. Lippincott & Co., of Philadelphia. The report on the progress of Pharmacy is an epitome of much useful knowledge, and is the more valuable from indicating the works that treat of the different articles that are passed in review. The Committee on the Drug Market, acting under instructions "to report on the fluctuations in the supply and demand of Drugs, their variations in quality and adulterations and sophistications," have made a report on their examination of a few articles of prime pharmaceutical and medicinal importance that will scarcely increase the confidence of physicians in the purity and activity of the medicines they prescribe. We quote the report on Sarsaparilla: "During the past year, the market has been repeatedly searched, in vain, for a single parcel, no matter how small, of the true Rio Negro Sarsaparilla. This, the most valuable, if not the only valuable variety of Sarsaparilla, has entirely disappeared from the market, and would probably be forgotten in the channels of trade, except that some of the makers of syrups and extracts still represent their preparations as being made from it."

Of seven specimens of Æther Fortior, two are reported as officinal and right in all important respects, and five as unfit for use. Of ten specimens of Chloroform, "not one was up to the standard in freedom from foreign compounds, though five were so nearly up to it as to be considered very good. The remainder were less clean, though, with one exception, not very unclean. One was very bad, unfit even for external use." The report concludes, "Neither the pharmaceutist nor the physician should ever permit chloroform to be used as an anæsthetic without first submitting it to the officinal tests." The "Proceedings" give us pleasing evidence of the vitality of the Association and of the good it is accomplishing to Pharmacy and Medicine.

---

*A Treatise on Human Physiology; Designed for the use of Students and Practitioners of Medicine.* By John C. Dalton, Jr., M. D. 3d Edition. Revised and Enlarged. Blanchard & Lea. 1864.

This work, recognized as a standard text-book by the medical schools, and with which the members of the profession are so familiar, demands but a brief notice. Its popularity is attested by the rapidity with which former editions have been exhausted. The following extract from the preface to the present edition indicates the changes that have been made in the work: "The improvements and additions which have been introduced consist in the incorporation into the text of certain new facts and discoveries, relating mainly to details which have made their appearance within the last three years. Such are the experiments of the author with regard to the secretion and properties of the parotid saliva in the human subject, and the quantative analysis of this fluid by Mr. Perkins; the valuable observations of Prof. Austin Flint, Jr., on Stercorine, Cholaterine, and the effects of permanent biliary fistula, and those of Prof. Jeffries Wyman on Fissure of Hare-lip in the Median Line, from arrest of development."

---

*Enlarged and Revised to* 1864. *The Medical Formulary.* Being a collection of Prescriptions, derived from the Writings and Practice of many of the most eminent Physicians in America and Europe, etc., etc., etc. By Benjamin Ellis, M. D. late Professor of Materia Medica and Pharmacy in the Philadelphia Collage of Pharmacy. Eleventh Edition. Carefully Revised and much Extended. By Robert P. Thomas, M. D., Professor of Materia Medica in the Philadelphia College of Pharmacy. "*Morbos andene non elequentia sed remedis curarè.*"—Cels. De Med , Lib. 1. Philadelphia. Blanchard & Lea. 1864.

We sometimes hear objections urged to the use of printed formulæ, as unworthy the attention of an educated physician; insisting that as the phases of disease are varied almost infinitely, so should the prescriptons vary also, and that this can only be done at the bed-side of the patient. This may be true to some extent, in theory, but in practice a large majority of the members of the profession frankly confess that the elegant and judicious formation of prescriptions is one of the difficulties which the young practitioner of medicine is obliged to encounter. And we are free to say that he must be a most accomplished physician who can glean no new or valuable idea from the "Medical Formulary."

That this work is highly esteemed by the profession throughout this country is manifest from the fact that we have before us the Eleventh Edition. The work was undertaken to aid the young practitioner in his first experiences, to furnish models for extemporaneous formulas, the proportions of which may be increased or diminished according to the age, sex, constitution or idiosyncrasy of the patient, all of which are to be taken into consideration, as well as the climate and peculiar epidemic which may be prevailing.

"It contains in a condensed form, and we think advantageously arranged, many of the most important prescriptions employed in modern practice."

"The application of remedies to diseases has been generally left to the judgment of the practitioner, and therapeutical detail as much as possible avoided, as it would have been inconsistent with the nature and design of the work."

The arrangement of the book is based on Chapman's old classification, so that we not only have elegant models of prescriptions furnished us, but these models presented in classes, which are of themselves a sort of suggestive system of Materia Medica.

We cordially endorse the favorable opinion which the profession has for so long a time entertained of this work, and commend it as worthy a place in the library of every physician.

---

*Medical Publications Received.*—

*Twenty-First Annual Report of the Managers of the State Lunatic Asylum for the Year* 1863. Transmitted to the Legislature of New York. 1864.

*Eleventh Annual Meeting for the Years* 1861–2, *and* 3, *of the Illinois State Medical Society.* Held at Jacksonville, May 5th, 1863.

*The New York Medical Independent and Pharmaceutical Reporter.* Weekly, at $2,00 per annum.

*The People's Journal of Health.* Edited by Justin Hayes, M. D., and C. R. Blackall, M. D. Chicago.

*Laryngoscopal Medications.* By Louis Elsbery, A. M., M. D.

